# Effects of atorvastatin on renal function in patients with dyslipidemia and chronic kidney disease: assessment of clinical usefulness in CKD patients with atorvastatin (ASUCA) trial

**DOI:** 10.1007/s10157-016-1304-6

**Published:** 2016-07-08

**Authors:** Genjiro Kimura, Masato Kasahara, Kenji Ueshima, Sachiko Tanaka, Shinji Yasuno, Akira Fujimoto, Toshiya Sato, Miyuki Imamoto, Shinji Kosugi, Kazuwa Nakao

**Affiliations:** 1 Asahi Rosai Hospital, Owariasahi, Aichi Japan; 2 Department of EBM Research, Institute of Advancement of Clinical and Translational Science, Kyoto University Hospital, Kyoto, Japan; 3 Institute for Clinical and Translational Science, Nara Medical University Hospital, 840 Shijo-cho, Kashihara, Nara, Japan; 4 Department of Biostatistics, Kyoto University School of Public Health, Kyoto, Japan; 5 Department of Food and Nutritional Science, Kobe Women’s Junior College, Kobe, Japan; 6 Department of Medical Ethics/Medical Genetics, Kyoto University School of Public Health, Kyoto, Japan; 7 Medical Innovation Center, Kyoto University Graduate School of Medicine, Kyoto, Japan

**Keywords:** Chronic kidney disease (CKD), Hyperlipidemia, Low-density lipoprotein cholesterol (LDL-C), Statins, Reno-protective effect

## Abstract

**Background:**

Dyslipidemia is a risk factor for the progression of chronic kidney disease (CKD). While conventional lipid lowering therapy provides a benefit to CKD management, the effect of statins on eGFR remains unclear.

**Methods:**

A prospective, multi-center, open-labeled, randomized trial. Total of 349 CKD patients with hyperlipidemia were randomized into 2 groups, and followed for 2 years. Group A included patients who were treated with atorvastatin. Group C were treated with conventional lipid lowering drugs other than statin. Primary endpoint was changes in eGFR. Secondary endpoints included changes in urinary albumin excretion, serum LDL-C, serum triglyceride, cardio-vascular events and all-cause mortality.

**Results:**

As the primary endpoint, eGFR decreased by 2.3 ml/min/1.73 m^2^ in Group A and by 2.6 ml/min/1.73 m^2^ in Group C, indicating that there was no difference in change of eGFR between the two groups. As secondary endpoints, atorvastatin succeeded to reduce serum LDL-C level significantly and rapidly, but conventional therapy did not. In fact, mean LDL-C level did not reach the target level of 100 mg/dl in Group C. Serum triglyceride was lowered only by atorvastatin, but not conventional drugs. The number of cardiovascular events and all-cause mortality did not differ between in two groups.

**Conclusion:**

The ASUCA (Assessment of Clinical Usefulness in CKD Patients with Atorvastatin) trial demonstrated that atorvastatin failed to exhibit reno-protections compared to conventional therapy in Japanese patients with dyslipidemia and CKD. It would be due in part to the ability of atorvastatin to more potently reduce serum LDL and triglycerides compared to conventional therapy.

## Introduction

An increased prevalence of chronic kidney disease (CKD) [1] could be a future burden in our society. We must take countermeasures to prevent such epidemic. The Treating to New Targets (TNT) study was sub-analyzed to examine if the atorvastatin treatment at dose of either 80 or 10 mg for approximately 5 years could provide benefits on renal function in patients with coronary heart disease, and the study found that an estimated glomerular filtration rate (eGFR) was improved by atorvastatin with both doses of atorvastatin [2]. Athyros’s research group performed the subanalysis of the GREACE study in patients with coronary artery disease and lipid abnormality. They found that statin treatment was associated with improving eGFR while such benefit was not observed in patients without statin [3]. Sandhu et al. also reported the positive effects of statins on renal function in their meta-analysis [4].

While it is assumed that atorvastatin could also exhibit reno-protective effects, a large-scale clinical study focusing on eGFR as primary endpoint has not been conducted. The LORD trial is considering the renal function as a primary endpoint, but the sample size is small [5]. Here, we conducted a large-scale clinical trial (ASUCA; assessment of clinical usefulness in CKD patients with atorvastatin) to investigate if atorvastatin could provide reno-protective effects in Japanese patients with CKD and dyslipidemia.

## Materials and methods

The rationale and design of the ASUCA trial have already been published. [6]. The ASUCA trial was a prospective, multi-center, open labeled, randomized trial performed in Japan. This study was registered at University Hospital Medical Information Network-Clinical Trials Registry (UMIN-CTR) under the trial identification number UMIN000001778 and has been approved by the Ethics Committee at the Kyoto University Graduate School of Medicine (C-271). The trial was conducted in accordance with the Declaration of Helsinki Principles.

### Participants

The inclusion criteria in this trial included fulfillment of all of the following at enrollment: subjects should be (1) 40 ≤ age < 75; (2) not treated with statins; (3) with positive proteinuria and eGFR ≥60 (ml/min/1.73 m^2^); (4) eGFR <60 ml/min/1.73 m^2^ at enrolment; (5) LDL-C ≥140 mg/dl in subjects not taking any dyslipidemia-treating agents or LDL-C ≥100 mg/dl in those taking dyslipidemia-treating agents other than statins.

The exclusion criteria were based on fulfillment of at least one of the following: (1) eGFR <30 ml/min/1.73 m^2^; (2) systolic blood pressure ≥180 mmHg or diastolic blood pressure ≥110 mmHg; (3) hemoglobin A1c (HbA1c) ≥8.5 %; (4) familial hypercholesterolemia; (5) secondary hypercholesterolemia including nephrotic syndrome; (6) liver dysfunction including acute hepatitis, chronic active hepatitis, liver cirrhosis, and hepatoma; (7) past history of severe side effects of atorvastatin; (8) pregnancy, possibility of pregnancy, or breast-feeding woman.

### Study design

After confirming the patient’s eligibility, each patient who provided written informed consent was randomly assigned to the Group C (diet therapy with non-statin treatment) or Group A (diet therapy and atorvastatin treatment). The following factors were used for stratified randomization: (1) gender, (2) hypertension, (3) diabetes mellitus, (4) treatment with renin angiotensin aldosterone system (RAAS) inhibitors. The target serum LDL-C level was <100 mg/dl. The follow-up period was 2 years. All patients basically received an adequate dietary advice of the non-face-to-face method. If dietary treatment in the Group C fails to reduce LDL-C level to the target level within the first 3 months, additional anti-dyslipidemic drugs except statins were allowed to be administered. The initial dose of atorvastatin in Group A was 10 mg/day and then adjusted to 5–20 mg/day. If the LDL-C level did not reduce to the target in the Group A, additional anti-dyslipidemic drugs except statins and fibrates were allowed to be used.

### Outcome measures

The primary outcome measure is the changes in eGFR (ml/min/1.73 m^2^) and based on serum creatinine measurement by the central laboratory. The secondary outcomes are (1) changes in urinary albumin/creatinine ratio (mg/g); (2) changes in serum LDL-C level; (3) changes in serum triglyceride level; (4) the number of total deaths, and (5) cerebro-cardiovascular events, which include cerebro-cardiovascular death and hospitalization due to cerebro-cardiovascular disease with revascularization, nonfatal cerebral bleeding and cerebral infarction, hemodialysis, and renal transplantation. Laboratory tests during a study period were performed at central laboratory (SRL, Inc., Tachikawa, Japan), and were scheduled to be done just before the start of treatment protocol, and 1, 3, 6, 9, 12, 18 and 24 months after the start of treatment protocol.

### Statistical considerations

The primary endpoint is a comparison of the changes in eGFR between the two arms after 2 years of treatment, using covariance analysis with stratification factors in randomization (sex, with or without hypertension, with or without diabetes mellitus, with or without RAAS inhibitors) as covariates. This analysis is performed based on the principal of intention-to-treat population. The effects of statins on eGFR reported in the GREACE study [3], the TNT study [2], the MEGA study [7], and meta-analyses [4] ranged between 1.9 and 7 ml/min/1.73 m^2^. Based on these studies [2–4, 7], we assume the standard deviation of changes per year in eGFR to be 12 ml/min/1.73 m^2^ in both groups and the difference between the two arms to be 4 ml/min/1.73 m^2^. To achieve a power of 80 %, a total of 286 patients were required. Assuming the uncertainty of setting of parameters, we planned to enroll 165 patients per arm.

## Results

### The flow chart of the ASUCA trial

**Fig. 1:**
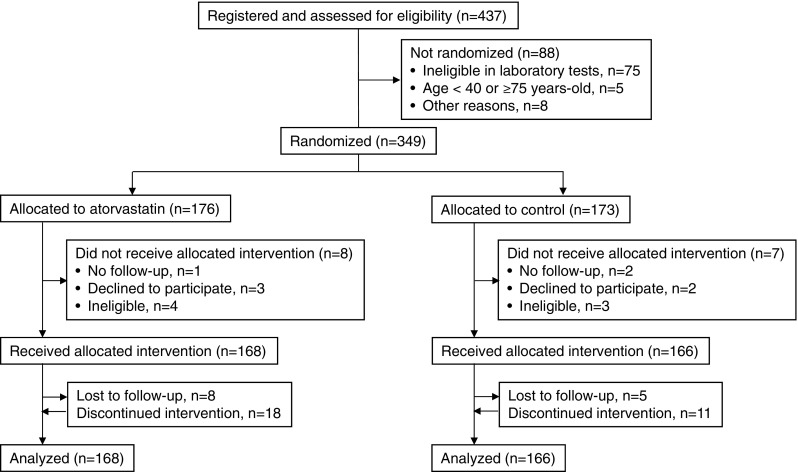
Patient’s flowchart in the ASUCA trial

Figure 1 shows patient’s flowchart of the ASUCA trial. All 437 patients were registered between April 2009 and March 2011. 88 patients were excluded by the assessment of eligibility. Among 437 patients registered, 349 were randomized after eligibility was assessed. The major reason for exclusion was ineligibility in laboratory tests before registration. Among the 349 patients, 15 were excluded due to no follow-up, declining to participate, and ineligibility found after randomization. Consequently, 334 patients were followed from January 2011 to May 2013 as FAS (full analysis set) population, consisted of 168 patients in the Group A and 166 patients in the Group C. Finally, PPS (per protocol set) population, consisted of 142 patients in the Group A and 150 patients in the Group C.

### Baseline

Tables 1 and 2 show baseline characteristics and laboratory tests of the 334 patients. In Group A and Group C, mean ages of subjects were 63.2 and 63.1 years, respectively. A percentage of hypertension at baseline was 60.7 % in Group A and 62.7 % in Group C. Prevalence of diabetic patients were 34.5 and 33.1 %. Past history of cerebro-cardiac diseases were within 10 %. Mean eGFR at baseline in Group A and Group C were 56.0 and 54.0 ml/min/1.73 m^2^. Mean LDL-C and log-transformed mean urinary albumin excretion were 142.2 and 145.9 mg/dl, 3.60 and 3.89 mg/g Cr.

**Table 1:** Patients’ characteristics

	*n* (%)/mean ± SD	
	Group A (*n* = 168)	Group C (*n* = 166)
Male	105 (62.5)	108 (65.1)
Age, years^a^	63.2 ± 7.9	63.1 ± 8.3
Disease complication (with duplication)		
Hypertension	102 (60.7)	104 (62.7)
Diabetes	58 (34.5)	55 (33.1)
Diabetic neuropathy	13 (7.7)	5 (3.0)
Diabetic retinopathy	11 (6.5)	11 (6.6)
Glomerulonephritis	18 (10.7)	20 (12.0)
Past history (with duplication)		
Cerebrovascular accident	8 (4.8)	11 (6.6)
Myocardial infarction	0 (0.0)	2 (1.2)
Angina pectoris	2 (1.2)	2 (1.2)
Heart failure	3 (1.8)	4 (2.4)
Arteriosclerosis obliterans	4 (2.4)	1 (0.6)
Normal ECG^b^	135 (80.8)	125 (76.2)
Smoker		
Current	19 (11.3)	28 (16.9)
Past	31 (18.5)	28 (16.9)
Alcohol drinker		
Current	70 (41.7)	64 (38.6)
Past	7 (4.2)	3 (1.8)

^a^Mean ± SD

^b^Normal electrocardiogram

**Table 2:** Baseline laboratory test

	Mean ± SD	
	Group A (*n* = 168)	Group C (*n* = 166)
eGFR^a^, ml/mm/1.73 m^2^	56.0 ± 11.6	54.0 ± 11.6
LDL-C, mg/dl	142.2 ± 26.7	145.9 ± 29.4
TG, mg/dl	172.1 ± 98.2	189.9 ± 145.2
HDL-C, mg/dl	52.6 ± 13.8	51.0 ± 12.1
U-Alb^b^, mg/g creatinine	248.1 ± 647.9	373.4 ± 842.1
Log-transformed U-Alb, mg/g creatinine	3.60 ± 1.95	3.89 ± 2.09
SBP, mmHg	134.2 ± 17.3	132.2 ± 15.3
DPB, mmHg	76.2 ± 10.9	77.4 ± 10.2
Heart rate, min^−1^	69.9 ± 11.0	72.3 ± 11.2
BMI, kg/m^2^	25.6 ± 3.4	25.6 ± 3.9

^a^Estimated glomerular filtration rate

^b^Urinary albumin excretion

### Concomitant treatment

Table 3 shows concomitant treatments during the study. Patients who took lipid lowering agents other than statin were one-quarters at baseline, 83.3 % at the end of follow-up in Group C. Ezetimibe accounted 72.0 % of the patients in Group C. Two-thirds were treated by RAAS inhibitors during the study.

**Table 3:** Concomitant treatment

	*n* (%)			
	Baseline		End of follow-up	
	Group A	Group C	Group A	Group C
Lipid lowering agents other than statin	36 (21.4 %)	40 (24.1 %)	7 (4.9 %)	125 (83.3 %)
Fibrate	13 (7.7 %)	17 (10.2 %)	0 (0.0 %)	26 (17.3 %)
Probucol	0 (0.0 %)	1 (0.6 %)	0 (0.0 %)	8 (5.3 %)
Ezetimibe	13 (7.7 %)	12 (7.2 %)	4 (2.8 %)	108 (72.0 %)
Resin	1 (0.6 %)	2 (1.2 %)	1 (0.7 %)	7 (4.7 %)
Others	9 (5.4 %)	14 (8.4 %)	2 (1.4 %)	24 (16.9 %)
Blood pressure lowering drugs	125 (74.4 %)	125 (75.3 %)	107 (75.4 %)	115 (76.7 %)
ARB^a^	96 (57.1 %)	105 (63.3 %)	83 (58.5 %)	94 (62.7 %)
ACE-I^b^	10 (6.0 %)	15 (9.0 %)	9 (6.3 %)	14 (9.3 %)
Diuretics	29 (17.3 %)	24 (14.5 %)	22 (15.5 %)	20 (13.3 %)
α blocker	4 (2.4 %)	6 (3.6 %)	5 (3.5 %)	5 (3.3 %)
β blocker	17 (10.1 %)	26 (15.7 %)	15 (10.6 %)	23 (15.3 %)
Calcium antagonists	76 (45.2 %)	73 (44.0 %)	67 (47.2 %)	68 (45.3 %)
Aldosterone antagonist	6 (3.6 %)	5 (3.0 %)	6 (4.2 %)	5 (3.3 %)
Others	3 (1.8 %)	2 (1.2 %)	2 (1.4 %)	2 (1.3 %)

^a^Angiotensin 2 receptor blockers

^b^Angiotensin converting enzyme inhibitor

### Changes in the lipid profile

**Fig. 2:**
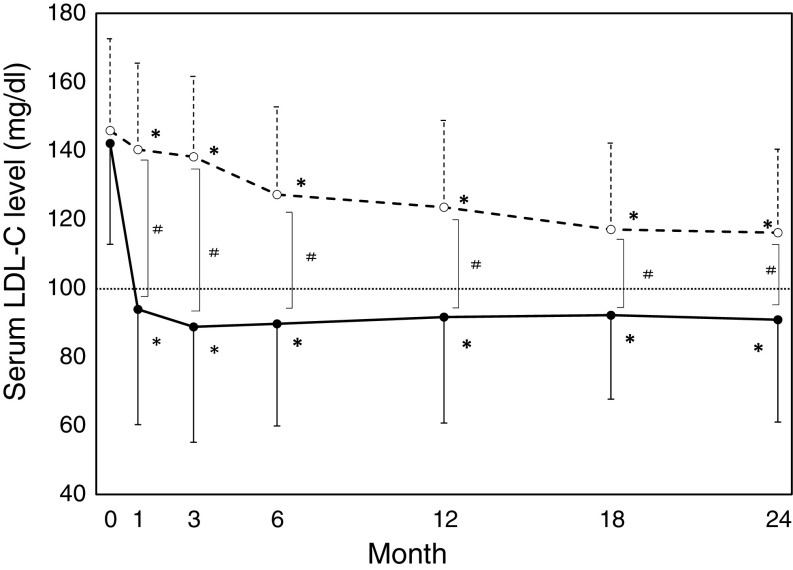
Time course of LDL-C concentration. *Solid line* and *dashed line* represent Group A (atorvastatin) and B (control), respectively. *Dotted line* represents recommended value of Japanese society of nephrology. *Error bars* represent standard deviation. *p < 0.05: each point value vs. baseline value, ^#^ *p* < 0.05: group A vs group C

**Fig. 3:**
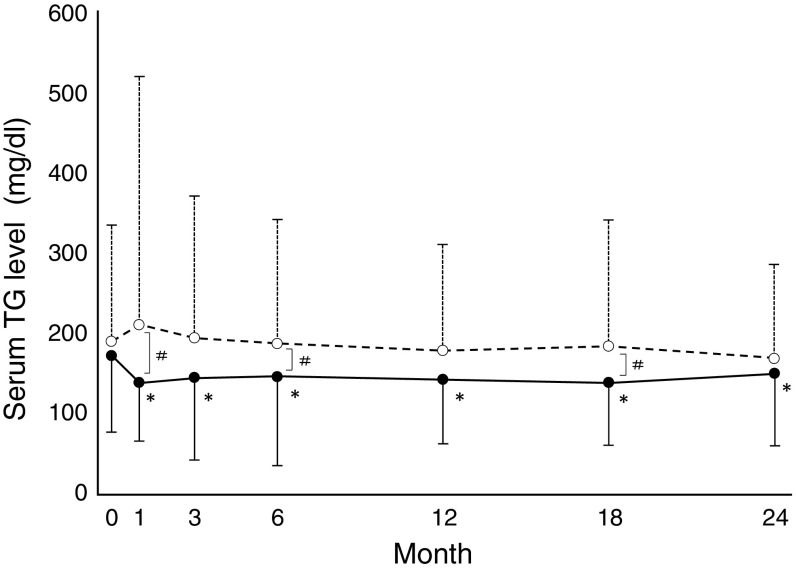
Time course of serum TG. *Solid line* and *dashed line* represent Group A (atorvastatin) and C (control), respectively. *Error bars* represent standard deviation. *p < 0.05: each points value vs. baseline value, ^#^ *p* < 0.05: Group A vs. Group C

The time course of LDL-C is shown in Fig. 2. LDL-C decreased significantly and rapidly in Group A and the level of LDL-C fulfilled the protocol requirement. The final average dosage of atorvastatin at the end of follow-up period was 10.5 mg. In contrast, the conventional therapy slowly but significantly decreased in serum LDL-C in Group C compared to Group A. In Group C, the LDL-C concentration ended up to 116.0 mg/dl. While this level, met recommendation of Japanese Society of Nephrology, it did not reach the target level of 100 mg/dl. TG was significantly reduced only by atorvastatin, but not conventional drugs (Fig. 3). Atorvastatin lowered serum HDL levels (−2.2 mg/dl), whereas control treatment reduced it by 2.9 mg/dl. The difference on the HDL lowering effects between two groups did not reach statistical significance. In addition, it was found that serum HDL levels were not associated with GFR in our study (data not shown).

### Changes in the GFR

**Fig. 4:**
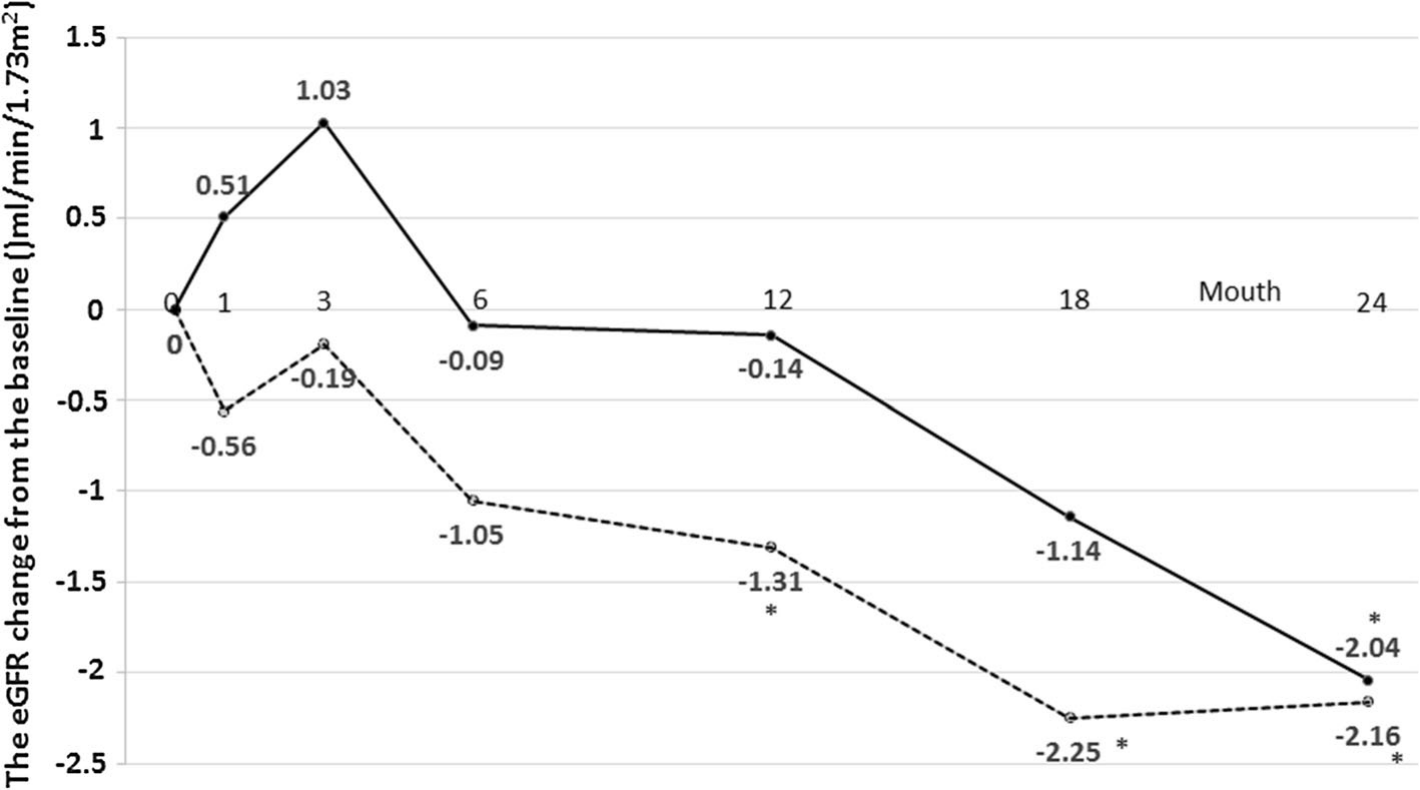
Time course of eGFR changes. *Solid line* and *dashed line* represent Group A (atorvastatin) and C (control), respectively. *p < 0.05: each point value vs. baseline value

**Fig. 5:**
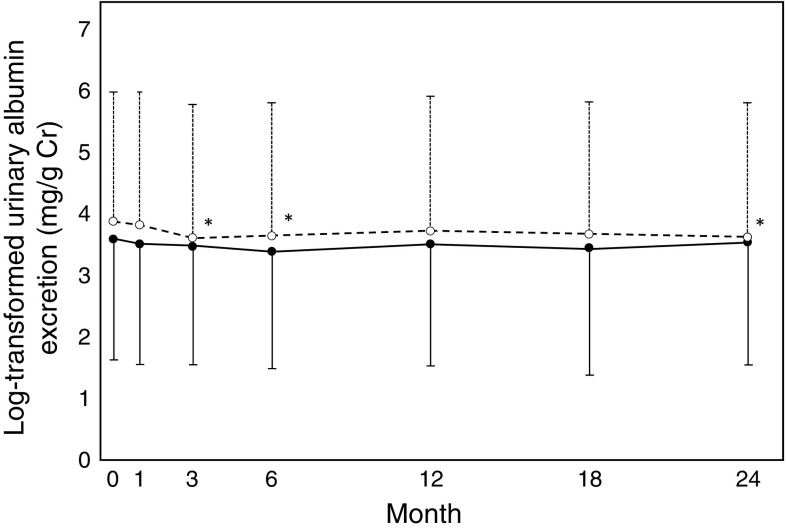
Time course of log-transformed urinary albumin excretion. *Solid line* and *dashed line* represent Group A (atorvastatin) and C (control), respectively. *Error bars* represent standard deviation. *p < 0.05: each point value vs. baseline value

As shown in Table 4, the changes in mean eGFR (−2.6 ml/min/1.73 m^2^) in Group C was similar to that (−2.3 ml/min/1.73 m2) in Group A. The difference in mean eGFR changes between two groups was not reach statistical significant (*p* = 0.851). Interestingly, a significant reduction of eGFR compared to baseline level was observed at 24 months in Group A, whereas it was detected from 12 month in Group C. A level of eGFR was identical between two groups at 24 month (Fig. 4). There was significant different in the changes of albuminuria in Group C (Fig. 5). There was no significant difference in composite of cardiovascular events and all-cause of death between Group A and Group C (Table 5). Prespecified subgroup analysis on change in eGFR is shown in Table 6. There was a statistically significant difference between Group A and Group C (*p* = 0.015) only in the subgroup of taking a lipid lowering drugs at enrollment.

**Table 4:** Changes in mean eGFR levels

Changes in eGFR	Mean ± SD	
	Group A (*n* = 133)	Group C (*n* = 146)
eGFR^a^ at baseline	56.2 ± 11.2	54.4 ± 11.6
eGFR after 2 years of treatment	53.9 ± 13.2	51.9 ± 13.7
Difference	−2.3 ± 8.7	−2.6 ± 8.8
Estimated difference (95 % CI)	0.19 (−1.85 to 2.24)	
*P* value	0.851	

^a^Estimated glomerular filtration rate

**Table 5:** The number of cerebro-cardiovascular events

	Group A (*n* = 168)	Group C (*n* = 166)	*p* value
Cardiovascular events	4 (2.4 %)	2 (1.2 %)	0.685
Sudden death	1 (0.6 %)	0 (0.0 %)	
AMI^a^	0 (0.0 %)	1 (0.6 %)	
AP^b^	1 (0.6 %)	0 (0.0 %)	
Stroke	2 (0.6 %)	0 (0.0 %)	
ESRD^c^	1 (0.6 %)	1 (0.6 %)	
All-cause death	1 (0.6 %)	1 (0.6 %)	1.000

^a^Acute myocardial infarction

^b^Angina pectoris

^c^End stage renal disease

**Table 6:** Prespecified subgroup analysis on change in eGFR

	*n*	Estimated difference	95 % CI	*p* value
Sex				
Male	213	−0.25	−2.91 to 2.39	0.847
Female	121	1.25	−1.91 to 4.43	0.434
Age, years				
<65	167	−0.37	−3.55 to 2.81	0.817
≥65	167	0.48	−2.17 to 3.14	0.717
BMI, kg/m^2^				
<25	168	0.63	−2.11 to 3.39	0.648
≥25	166	−0.16	−3.24 to 2.9	0.914
HDL-C, mg/dl				
≥40 (50: female)	238	0.42	−1.93 to 2.78	0.722
<40 (50: female)	85	−0.38	−4.62 to 3.85	0.857
LDL-C, mg/dl				
<140	142	0	−3.06 to 3.05	0.999
≥140	181	0.52	−2.21 to 3.26	0.706
TG, mg/dl				
<150	148	0.99	−1.96 to 3.95	0.506
≥150	175	−0.52	−3.39 to 2.35	0.719
U-Alb^a^, mg/g creatinine				
<30	168	−0.02	−2.8 to 2.75	0.986
≥30	154	0	−2.96 to 2.96	0.999
U-Alb, mg/g creatinine				
<300	256	0.24	−1.84 to 2.33	0.819
>300	66	−1.52	−7.02 to 3.98	0.581
eGFR^b^, ml/min/1.73^2^				
≥45	263	0.36	−1.71 to 2.44	0.727
<45	60	1.22	−5.59 to 8.03	0.719
hs CRP^c^, ng/ml				
<634 (median)	161	0.51	−2.12 to 3.15	0.698
≥634 (median)	162	−0.28	−3.42 to 2.84	0.856
Diabetes				
No	221	0.46	−1.76 to 2.69	0.682
Yes	113	−0.06	−4.22 to 4.13	0.977
Hypertension				
No	128	−0.17	−3.32 to 2.98	0.917
Yes	206	0.49	−2.20 to 3.18	0.720
LVH^d^				
No	310	0.27	−1.81 to 2.36	0.796
Yes	21	−3.21	−17.57 to 11.13	0.619
History of CVD^e^				
No	277	0.08	−2.08 to 2.24	0.94
Yes	57	0.46	−5.49 to 6.43	0.874
Lipid lowering drugs at enrollment				
No	258	−1.31	−3.56 to 0.93	0.249
Yes	76	5.68	1.11 to 10.25	0.015
RAAS inhibitor^f^ at enrollment				
No	116	0	−3.59 to 3.59	0.998
Yes	218	0.28	−2.27 to 2.85	0.824

^a^Urinary albumin excretion

^b^Estimated glomerular filtration rate

^c^High sensitivity c-reactive protein

^d^Left ventricular hypertrophy

^e^Cardio vascular disease

^f^Renin angiotensin aldosterone system inhibitor

## Discussion

Statin might protect kidney in addition to lowering serum cholesterol level. Although precise mechanisms for its reno-protection remains unclear, one of the potential mechanisms could be an increase in endothelial NO production [8]. A reduction in vascular resistance [9] and increase in renal blood flow with higher cardiac output [10] might be accounted for by such increase in endothelial NO. Blocking mesangial proliferation [11, 12] and stabilizing vascular plaques [13, 14] by statin also likely contribute to slow the progression of renal disease. Among several types of statins, atorvastatin, is a lipid-soluble type statin, might be more potent to block the development of kidney disease. In fact, a recent study has demonstrated that atorvastatin was able to improve eGFR in patients with diabetes and/or cerebro-cardiovascular disease [3, 4]. But these previous reports targeted patients with only severe diabetes and/or cerebro-cardiovascular disease. It is also very important to investigate patients with less risk for these diseases. Here, the ASUCA trial was conducted to examine if atorvastatin could be more protective than other conventional therapy other than statins in preventing the progression of renal disease in Japanese patients with CKD and hyperlipidemia. There was no significant difference in eGFR at the time after 24 months.

Lipid lowering effect of atorvastatin seems more potent than that of conventional therapy as it took just 1 month for atorvastatin to reduce serum LDL to the target level in Group A. Likewise, atorvastatin treatment, as opposed to conventional therapy, was able to reduce serum triglyceride level significantly. Thus, we expected that atorvastatin might be more protective in renal function.

However, the effect of atorvastatin did not show a better renal protection at the time after 24 months compared to conventional treatment. De Zeeuw et al. suggested that some protective effect of atorvastatin on the renal function [15] while the ASUCA trial did not show the superior effect of atorvastatin to conventional treatment in terms of renal function for less risk patients. The background of subjects could be the reason of failure of atorvastatin to show beneficial effect. In the ASUCA trial, less than 10 % of our patients have cerebro-cardiovascular disease compared to the TNT and GREACE study with 100 % subject with this disease. Approximately 30–35 % of subject has diabetes in our study while the CARDS study fulfills the entry criteria with diabetes [3, 16]. In addition, 70 % of patients were taking an established renal protective drug of RAAS inhibitors in our study. In turn, 79 % of patients in Group C had been administered ezetimibe. Since ezetimibe would have renal protective effect [17, 18], it is likely that ezetimibe might be reno-protective as much as atorvastatin in this study [19, 20].

It is interesting that Group C exhibited less GFR reduction after 18 months while Group A still showed the progressive decline at that point. While the precise mechanism remains unclear, it is likely that a less reduction in GFR in control group could be attributed to the beneficial effects from lipid lowering drugs. In our protocol, subjects in Group C, but not in Group A were allowed to take any lipid lowering drugs other than statin after 3 months. And treatment rate with other lipid lowering drugs were significantly higher at the end of study compared to that at baseline in Group C (Table 3). Since it has been shown that lipid lowering drugs exhibit the renoprotective effects, it is likely that using lipid lowering drugs was associated with a slowing down of GFR reduction in control group.

In conclusion, atorvastatin did not show reno-protective effect compare to conventional therapy for Japanese patients. The limitation of this study is that we could not compare the placebo control despite of the ethical issue. So, further investigation is needed to examine the effect of LDL lowering therapy on the reno-protection.

## References

[1] El-Atat FA, Stas SN, McFarlane SI, Sowers JR (2004). The relationship between hyperinsulinemia, hypertension and progressive renal disease. J Am Soc Nephrol.

[2] Shepherd J, Kastelein JJ, Bittner V, Deedwania P, Breazna A, Dobson S, Wilson DJ, Zuckerman A, Wenger NK, Treating to New Targets Investigators (2007). Effect of intensive lipid lowering with atorvastatin on renal function in patients with coronary heart disease: the treating to new targets (TNT) study. Clin J Am Soc Nephrol..

[3] Athyros VG, Mikhailidis DP, Liberopoulos EN, Kakafika AI, Karagiannis A, Papageorgiou AA, Tziomalos K, Ganotakis ES, Elisaf M (2007). Effect of statin treatment on renal function and serum uric acid levels and their relation to vascular events in patients with coronary heart disease and metabolic syndrome: a subgroup analysis of the GREek Atorvastatin and Coronary heart disease Evaluation (GREACE) Study. Nephrol Dial Transpl.

[4] Sandhu S, Wiebe N, Fried LF, Tonelli M (2006). Statins for improving renal outcomes: a meta-analysis. J Am Soc Nephrol.

[5] Fassett RG, Robertson IK, Ball MJ, Geraghty DP, Coombes JS (2010). Effect of atorvastatin on kidney function in chronic kidney disease: a randomized double-blind placebo-controlled trial. Atherosclerosis..

[6] Ueshima K, Kasahara M, Koya D, Babazono T, Sato T, Imamoto M, Yasuno S, Fujimoto A, Tanaka S, Kimura G, Nakao K (2013). Effects of atorvastatin on renal function in patients with dyslipidemia and chronic kidney disease: rationale and design of the ASsessment of clinical Usefulness in CKD patients with Atorvastatin (ASUCA) trial. Clin Exp Nephrol..

[7] Nakamura H, Mizuno K, Ohashi Y, Yoshida T, Hirao K, Uchida Y, MEGA Study Group (2009). Pravastatin and cardiovascular risk in moderate chronic kidney disease. Atherosclerosis..

[8] Laufs U, Gertz K, Huang P, Nickenig G, Bohm M, Dirnagl U, Endres M (2000). Atorvastatin upregulates type III nitric oxide synthase in thrombocytes, decreases platelet activation, and protects from cerebral ischemia in normocholesterolemic mice. Stroke.

[9] Cohn JN, Wilson DJ, Neutel J, Houston M, Weinberger MH, Grimm R, Smith DH, Sun W (2009). Coadministered amlodipine and atorvastatin produces early improvements in arterial wall compliance in hypertensive patients with dyslipidemia. Am J Hypertens.

[10] Sola S, Mir MQ, Lerakis S, Tandon N, Khan BV. Atorvastatin improves left ventricular systolic function and serum markers of inflammation in nonischemic heart failure. J Am Coll Cardiol. 2006; 17; 47(2):332–7. 10.1016/j.jacc.2005.06.088 16412856

[11] Katzir Z, Leibovitch E, Vaknin H, Schreiber L, Berger E, Matas Z, Fux A, Boaz M, Briliant A, Biro A (2013). Effect of atorvastatin on IgA nephropathy in the rat. Clin Nephrol.

[12] Shibata T, Tamura M, Kabashima N, Serino R, Tokunaga M, Matsumoto M, Miyamoto T, Miyazaki M, Furuno Y, Takeuchi M, Abe H, Okazaki M, Otsuji Y. Fluvastatin attenuates IGF-1-induced ERK1/2 activation and cell proliferation by mevalonic acid depletion in human mesangial cells. Life Sci. 2009; 22; 84(21–22):725–31. 10.1016/j.lfs.2009.02.022 19254730

[13] Shimojima M, Kawashiri MA, Nitta Y, Yoshida T, Katsuda S, Kaku B, Taguchi T, Hasegawa A, Konno T, Hayashi K, Yamagishi M (2012). Rapid changes in plaque composition and morphology after intensive lipid lowering therapy: study with serial coronary CT angiography. Am J Cardiovasc Dis..

[14] Bustos C, Hernandez-Presa MA, Ortego M, Tunon J, Ortega L, Perez F, Díaz C, Hernández G, Egido J (1998). HMG-CoA reductase inhibition by atorvastatin reduces neointimal inflammation in a rabbit model of atherosclerosis. J Am Coll Cardiol.

[15] De Zeeuw D, Anzalone DA, Cain VA, Cressman MD, Heerspink HJ, Molitoris BA, Monyak JT, Parving HH, Remuzzi G, Sowers JR, Vidt DG (2015). Renal effects of atorvastatin and rosuvastatin in patients with diabetes who have progressive renal disease (PLANET I): a randomised clinical trial. Lancet Diabetes Endocrinol..

[16] Colhoun HM, Betteridge DJ, Durrington PN, Hitman GA, Neil HA, Livingstone SJ, Charlton-Menys V, DeMicco DA, Fuller JH, CARDS Investigators (2009). Effects of atorvastatin on kidney outcomes and cardiovascular disease in patients with diabetes: an analysis from the collaborative atorvastatin diabetes study (CARDS). Am J Kidney Dis.

[17] Tamura Y, Murayama T, Minami M, Matsubara T, Yokode M, Arai H (2012). Ezetimibe ameliorates early diabetic nephropathy in db/db mice. J Atheroscler Thromb..

[18] Mori Y, Hirano T (2012). Ezetimibe alone or in combination with pitavastatin prevents kidney dysfunction in 5/6 nephrectomized rats fed high-cholesterol. Metabolism..

[19] Morita T, Morimoto S, Nakano C, Kubo R, Okuno Y, Seo M, Someya K, Nakahigashi M, Ueda H, Toyoda N, Kusabe M, Jo F, Takahashi N, Iwasaka T, Shiojima I (2014). Renal and vascular protective effects of ezetimibe in chronic kidney disease. Intern Med.

[20] Kouvelos GN, Arnaoutoglou EM, Milionis HJ, Raikou VD, Papa N, Matsagkas MI (2015). The effect of adding ezetimibe to rosuvastatin on renal function in patients undergoing elective vascular surgery. Angiology..

